# Clinicopathological Patterns, Associations, and Management of Orbital Involvement in Sinonasal Malignancies: A Retrospective Study

**DOI:** 10.7759/cureus.111067

**Published:** 2026-06-18

**Authors:** Muhammad A Zahid, Saleh Khurshied, Amina Sarfraz, Humna Rashid, Mehrun Nisa, Quratulain Zahra, Hira G Shah

**Affiliations:** 1 Ophthalmology, Monash Health, Clayton, AUS; 2 Otolaryngology - Head and Neck Surgery, Pakistan Institute of Medical Sciences, Islamabad, PAK; 3 Medicine and Surgery, Federal Medical College, Islamabad, PAK; 4 Medicine and Surgery, Pakistan Institute of Medical Sciences, Islamabad, PAK; 5 General Physician, Best Care Hospital, Chakwal, Islamabad, PAK; 6 Ophthalmology, Alshifa Trust Eye Hospital, Rawalpindi, PAK

**Keywords:** head and neck oncology, orbital exenteration, orbit metastasis, sinonasal tumor pathology, squamous cell carcinoma (scc)

## Abstract

Background

Sinonasal malignancies are uncommon head and neck tumors that frequently present at an advanced stage due to their deep anatomical location and nonspecific early symptoms. Orbital involvement represents an important indicator of locally aggressive disease and has direct implications for staging and treatment planning. This study was conducted to analyze the clinicopathological profile, radiological patterns of orbital invasion, and management strategies in patients with sinonasal malignancies involving the orbit.

Methods

A retrospective observational study was carried out in the Department of ENT - Head and Neck Surgery at the Pakistan Institute of Medical Sciences, Islamabad, Pakistan. Patients with histopathologically confirmed sinonasal malignancies and radiological evidence of orbital extension from April 2022 to March 2026 were included. Relevant demographic, histological, staging, orbital invasion patterns, treatment modalities, and discharge outcomes were extracted from hospital records. Data were analyzed using descriptive statistics, and associations were assessed using chi-square and Fisher’s exact tests where appropriate.

Results

A total of 78 patients were included. The mean age was 57.6 ± 12.2 years, and 45 (57.69%) were male. Squamous cell carcinoma was the most common histological subtype (28, 35.90%), followed by adenocarcinoma (18, 23.08%) and sinonasal undifferentiated carcinoma (13, 16.67%). All patients presented with advanced disease, including 16 (20.51%) at stage III and 62 (79.49%) at stage IV. Eye-related clinical features were common, with ocular pain/discomfort in 56 (71.79%), proptosis in 48 (61.54%), and epiphora in 44 (56.41%) patients.

A significant difference in mean age was observed across histological subtypes (p = 0.003), while no significant associations were found with gender (p = 0.92), stage (p = 0.41), or pattern of orbital invasion (p = 0.95). The highest level of erosion seen on radiology showed that only lamina papyracea erosion was the most frequent orbital involvement (31, 39.74%), followed by periorbital tissue (18, 23.08%), extraconal fat (13, 16.67%), extraocular muscles (10, 12.82%), and orbital apex (6, 7.69%). Orbit-preserving surgery was performed in 53 (67.95%) patients, while 16 (20.51%) underwent exenteration and 9 (11.54%) received chemoradiotherapy. A significant association was noted between histological subtype and surgical management (p < 0.001), with higher exenteration rates in sinonasal undifferentiated carcinoma and adenoid cystic carcinoma.

Conclusion

Sinonasal malignancies with orbital involvement most commonly presented at an advanced stage (predominantly stage IV), with squamous cell carcinoma being the most frequent histological subtype and the lamina papyracea being the commonest route of orbital extension. A significant variation in mean age was observed across histological subtypes, whereas no significant association was found between histology and gender, stage, pattern of orbital invasion, or overall treatment modality. However, histological subtype showed a significant association with surgical management, particularly the need for orbital exenteration.

## Introduction

Sinonasal malignancies are uncommon, representing approximately 3-5% of all head and neck cancers; however, they pose considerable diagnostic and therapeutic difficulties owing to the intricate anatomy of the sinonasal region and its close relationship with vital structures such as the orbit and skull base [1]. In their early stages, these tumors frequently remain clinically silent, and patients often present with advanced disease because of vague, nonspecific symptoms that lead to delayed diagnosis [2].

Orbital involvement is a well-established characteristic of advanced sinonasal malignancies and serves as an important factor in staging, treatment planning, and prognosis [3]. The medial orbital wall (lamina papyracea), due to its extremely thin bony architecture, is especially prone to early erosion, permitting tumor spread from the ethmoid and maxillary sinuses into the orbital cavity [4]. The extent of orbital invasion may vary from simple bony wall destruction to infiltration of orbital fat, extraocular muscles, and the orbital apex, with increasing involvement being associated with poorer functional outcomes [5]. From a clinical perspective, orbital invasion may manifest as proptosis, diplopia, ocular discomfort, restriction of eye movements, and visual deficits, typically reflecting advanced disease at the time of presentation [6]. Traditionally, orbital involvement was regarded as an indication for radical surgical procedures such as orbital exenteration; however, progress in imaging modalities, surgical techniques, and multimodal therapy has gradually shifted management toward orbit-preserving approaches in appropriately selected patients without compromising oncologic outcomes [7].

Computed tomography (CT) and magnetic resonance imaging (MRI) are pivotal in precisely delineating the extent of orbital and skull base involvement, thereby assisting in staging and surgical decision-making [8]. Additionally, the incorporation of endoscopic skull base surgery, induction chemotherapy, and adjuvant radiotherapy has enhanced the possibility of organ preservation in suitably selected cases [9]. Despite these improvements, sinonasal malignancies with orbital extension continue to present at advanced stages in the majority of patients, contributing to unfavorable prognosis and challenging management decisions [10]. Moreover, data from tertiary care centers in developing countries remain scarce, particularly regarding patterns of orbital invasion and treatment strategies derived from real-world clinical practice.

Accordingly, the present study aimed to evaluate the clinicopathological characteristics of sinonasal malignancies with orbital involvement, with special emphasis on histological distribution, demographic profile, and radiological patterns of orbital invasion. In addition, the study assessed the relationship between tumor histology, patterns of orbital involvement, and treatment modalities used in routine clinical practice using appropriate statistical methods. No survival or long-term outcome analysis was performed due to the retrospective nature of the study and the lack of follow-up data.

## Materials and methods

Study design, setting, and population

This retrospective observational study was conducted to evaluate the clinicopathological features, patterns of orbital involvement, and treatment strategies in patients with sinonasal malignancies. The study was carried out in the Department of ENT-Head and Neck Surgery at the Pakistan Institute of Medical Sciences, Islamabad, Pakistan. Medical records of patients managed between April 2022 and March 2026 were reviewed. Patients with histopathologically confirmed sinonasal malignancies and radiological evidence of orbital involvement on CT or MRI were included. Only consecutive cases with complete clinical documentation in hospital records and discharge files were analyzed.

Inclusion and exclusion criteria

Patients of all age groups and both genders were eligible if they had a confirmed histopathological diagnosis of sinonasal malignancy along with radiological, operative, or documented evidence of orbital involvement. Inclusion required complete clinical, radiological, and treatment records. Patients with benign lesions, recurrent tumors treated at external centers with incomplete documentation, and cases lacking adequate information regarding orbital status were excluded from the study.

Data source, variables, and data collection procedure

Data were obtained from hospital medical records, discharge summaries, radiology reports (CT/MRI), histopathology reports, and operative findings. The collected variables included demographic characteristics (age and sex), histological subtype, AJCC tumor stage, and highest extent of orbital involvement (lamina papyracea, periorbita, extraconal fat, extraocular muscles, and orbital apex). Treatment-related information included surgical approach (orbit-sparing surgery or orbital exenteration), radiotherapy, chemotherapy, and combined chemoradiotherapy. Orbital invasion was classified based on imaging findings, with confirmation from operative and clinical documentation, and the highest extent of erosion was recorded. Patients were grouped according to histological subtype for comparative statistical analysis.

Statistical analysis

Data were entered into Microsoft Excel (Microsoft Corp., Redmond, WA) and analyzed using SPSS Version 25 (IBM Corp., Armonk, NY). Categorical variables were summarized as frequencies and percentages, whereas continuous variables were presented as mean ± standard deviation.

Comparisons of mean age across histological groups were performed using one-way ANOVA. Associations between categorical variables, including histological subtype, sex distribution, stage at presentation, orbital invasion pattern, and treatment modality, were assessed using the chi-square test or Fisher’s exact test with Monte Carlo simulation when expected cell counts were low.

A p-value of less than 0.05 was considered statistically significant. The final analysis included evaluation of associations between histological subtype and clinicopathological variables as per revised study objectives. Survival analysis was not performed due to the lack of longitudinal follow-up data.

Ethical considerations

Approval for the use of patient records was obtained from the Head of the Department. As this was a retrospective study, informed consent was waived. Patient confidentiality was strictly maintained, and all data were anonymized prior to analysis.

## Results

A total of 78 patients with sinonasal malignancies involving the orbit were analyzed in this study. Squamous cell carcinoma represented the most frequent histopathological subtype, 28 (35.90%), followed by adenocarcinoma, 18 (23.08%), sinonasal undifferentiated carcinoma, 13 (16.67%), olfactory neuroblastoma, 9 (11.54%), adenoid cystic carcinoma, 7 (8.97%), and other rare tumor types, 3 (3.85%). The mean age of participants was 57.6 ± 12.2 years, and the mean age at presentation varied across histological categories, with the youngest patients observed in the olfactory neuroblastoma group (41.5 ± 8.7 years) and the oldest in the “other” malignancy group (76.5 ± 5.2 years). A statistically significant difference in age distribution was identified among the different histological subtypes (p = 0.003).

Male patients were slightly more common across all tumor categories; however, no statistically significant relationship was found between sex and histological subtype (p = 0.92). In addition, most patients presented with advanced disease, with stage IV accounting for 79.49% of cases across all histologies. There was no statistically significant association between tumor type and stage at presentation (p = 0.41), indicating a uniformly advanced disease profile at diagnosis regardless of histological subtype. These findings have been presented in detail in Table 1.

**Table 1:** Histological subtypes and clinicodemographic characteristics of patients with sinonasal malignancies Total number of cases (N): 78 (100%) *One-way ANOVA test **Fisher’s exact test (Monte Carlo simulation where appropriate) Staging as per the American Joint Committee on Cancer [11] SD, standard deviation; SNUC, sinonasal undifferentiated carcinoma

Histological Type	N (%)	Mean Age ± SD (years)	Male, N (%)	Female, N (%)	Stage III, N (%)	Stage IV, N (%)
Squamous cell carcinoma	28 (35.90)	58.0 ± 9.6	16 (57.14)	12 (42.86)	3 (10.71)	25 (89.29)
Adenocarcinoma	18 (23.08)	58.0 ± 10.2	10 (55.56)	8 (44.44)	3 (16.67)	15 (83.33)
SNUC	13 (16.67)	48.0 ± 11.1	7 (53.85)	6 (46.15)	3 (23.08)	10 (76.92)
Olfactory neuroblastoma	9 (11.54)	41.5 ± 8.7	5 (55.56)	4 (44.44)	2 (22.22)	7 (77.78)
Adenoid cystic carcinoma	7 (8.97)	68.0 ± 7.8	6 (85.71)	1 (14.29)	4 (57.14)	3 (42.86)
Other	3 (3.85)	76.5 ± 5.2	2 (66.67)	1 (33.33)	1 (33.33)	2 (66.67)
Total	78 (100)	57.6 ± 12.2	45 (57.69)	33 (42.31)	16 (20.51)	62 (79.49)
P-value	-	0.003*	0.92**	0.92**	0.41**	0.41**

**Figure 1:**
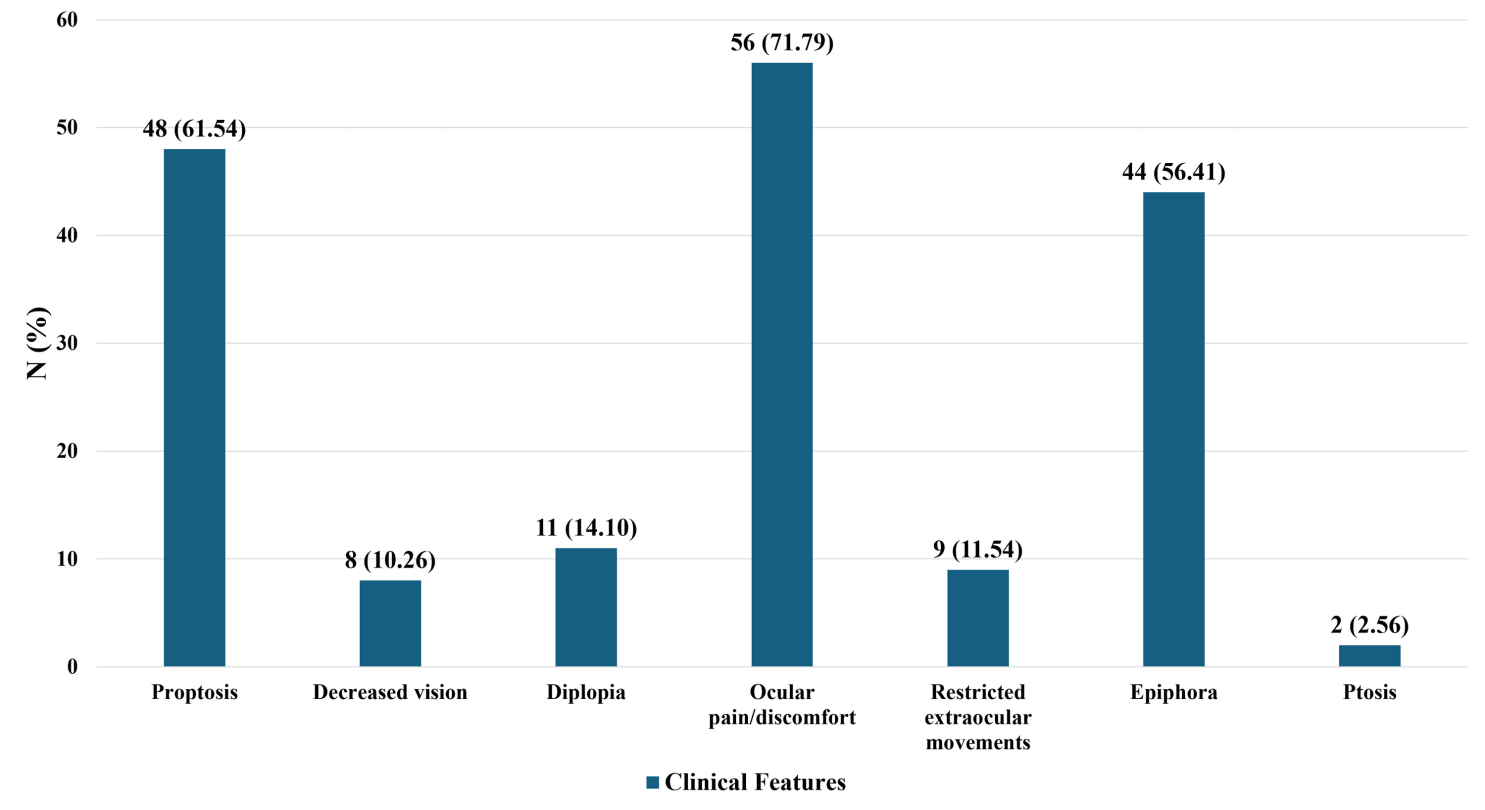
Eye-related clinical presentations in patients with sinonasal malignancies N: Number of patients Total number of cases: 78 Patients may present with more than one clinical feature; percentages are not mutually exclusive.

Eye-related clinical manifestations were frequently observed in patients with sinonasal malignancies involving the orbit. The most common symptom was ocular pain or discomfort 56 (71.79%), followed by proptosis 48 (61.54%) and epiphora 44 (56.41%). Diplopia was reported in 14.10% of patients, while restricted extraocular movements were present in 11.54%. Decreased vision was noted in 10.26% of cases, and ptosis was the least frequent finding (2.56%). These findings are illustrated in Figure 1. Patients often presented with more than one ocular symptom, reflecting variable degrees of orbital involvement at presentation.

The distribution of orbital anatomical site involvement showed that the lamina papyracea was the most frequently affected site (31/78, 39.74%), followed by the periorbita (18/78, 23.08%) and extraconal fat (13/78, 16.67%). Less frequent involvement was observed in the extraocular muscles (10/78, 12.82%), while the orbital apex was least commonly involved (6/78, 7.69%). Overall, the association between histological type and anatomical site distribution was not statistically significant on chi-square testing (p = 0.95), as detailed in Table 2.

**Table 2:** Association between histological subtype and pattern of orbital invasion in sinonasal malignancies Total number of cases (N): 78 (100%) SD, standard deviation; SNUC, sinonasal undifferentiated carcinoma

Histological Type	Lamina Papyracea, N (%)	Periorbita, N (%)	Extraconal Fat, N (%)	Extraocular Muscles, N (%)	Orbital Apex, N (%)	Total, N (%)
Squamous cell carcinoma	12 (15.38)	7 (8.97)	4 (5.13)	3 (3.85)	2 (2.56)	28 (35.90)
Adenocarcinoma	8 (10.26)	5 (6.41)	2 (2.56)	2 (2.56)	1 (1.28)	18 (23.08)
SNUC	5 (6.41)	3 (3.85)	2 (2.56)	2 (2.56)	1 (1.28)	13 (16.67)
Olfactory neuroblastoma	3 (3.85)	2 (2.56)	2 (2.56)	1 (1.28)	1 (1.28)	9 (11.54)
Adenoid cystic carcinoma	2 (2.56)	1 (1.28)	1 (1.28)	2 (2.56)	1 (1.28)	7 (8.97)
Other	1 (1.28)	0 (0.00)	2 (2.56)	0 (0.00)	0 (0.00)	3 (3.85)
Total	31 (39.74)	18 (23.08)	13 (16.67)	10 (12.82%)	6 (7.69%)	78 (100%)
P-value (chi-square test)	0.95					

Out of 78 patients, 69 underwent surgery. Among them, 53 (76.81%) patients had orbit-sparing surgery, while 16 (23.19%) patients required orbital exenteration. The remaining nine (11.54% of the total) patients had unresectable disease and were treated with definitive chemoradiotherapy, as detailed in Table 3.

**Table 3:** Treatment modalities in patients with sinonasal malignancies and orbital involvement Total number of cases (N): 78 (100%)

Treatment Modality	N (%)
Surgery alone	3 (3.85)
Surgery + radiotherapy	39 (50.00)
Surgery + chemoradiotherapy	27 (34.61)
Chemoradiotherapy alone	9 (11.54)
Total	78 (100)
Surgical approach	
Orbital-sparing surgery	53 (76.81)
Orbital exenteration	16 (23.19)
Total	69 (100)

A statistically significant association was observed between histological type and surgical management (Fisher’s exact test with Monte Carlo simulation, p < 0.001). Orbit-sparing surgery was the predominant treatment across all histological categories, including squamous cell carcinoma, 20/28 (71.43%), adenocarcinoma, 14/18 (77.78%), sinonasal undifferentiated carcinoma, 8/13 (61.54%), olfactory neuroblastoma, 6/9 (66.67%), adenoid cystic carcinoma, 4/7 (57.14%), and other tumors, 1/3 (33.33%).

The proportion of orbital exenteration varied across histologies, being 28.57% in squamous cell carcinoma, 22.22% in adenocarcinoma, 38.46% in sinonasal undifferentiated carcinoma, 33.33% in olfactory neuroblastoma, 42.86% in adenoid cystic carcinoma, and 66.67% in other tumors. These findings demonstrate a statistically significant relationship between histological subtype and the choice of surgical approach, as detailed in Table 4.

**Table 4:** Association between histological type and surgical management *Fisher’s exact test (Monte Carlo simulation) Total number of cases (N): 78 (100%) SNUC, sinonasal undifferentiated carcinoma

Histological Type	Orbit-Sparing Surgery, N (%)	Orbital Exenteration, N (%)	Total, N (%)
Squamous cell carcinoma	20 (71.43)	8 (28.57)	28 (35.90)
Adenocarcinoma	14 (77.78)	4 (22.22)	18 (23.08)
SNUC	8 (61.54)	5 (38.46)	13 (16.67)
Olfactory neuroblastoma	6 (66.67)	3 (33.33)	9 (11.54)
Adenoid cystic carcinoma	4 (57.14)	3 (42.86)	7 (8.97)
Other	1 (33.33)	2 (66.67)	3 (3.85)
Total	53 (67.95)	16 (20.51)	78 (100)
P-value	< 0.001*		

## Discussion

Sinonasal malignancies with orbital extension represent advanced head and neck cancers that typically present late due to non-specific early symptoms. In this retrospective study, 78 patients were analyzed. The mean age was 57.6 ± 12.2 years, with a male predominance, which is consistent with previously reported demographic patterns showing peak incidence in the fifth to seventh decades of life and a similar gender distribution, likely related to environmental and occupational exposure [1,2].

Histologically, squamous cell carcinoma was the most frequent subtype (35.90%), followed by adenocarcinoma, sinonasal undifferentiated carcinoma, and other rare tumors. This distribution is in agreement with earlier reports where epithelial malignancies, particularly squamous cell carcinoma, constitute the majority of sinonasal cancers involving the orbit [1,3].

Orbital invasion most commonly involved the lamina papyracea (39.74%), followed by periorbital tissues and extraconal fat, while orbital apex involvement was least frequent. This pattern reflects the anatomical route of least resistance for tumor spread, as the lamina papyracea represents a thin bony barrier between the sinonasal cavity and orbit. Similar findings have been reported by Turri-Zanoni et al., who emphasized stepwise orbital extension as a key feature of disease progression [4,12].

All patients presented with advanced disease, with 79.49% in stage IV, consistent with prior studies highlighting delayed diagnosis due to non-specific early sinonasal symptoms and deep anatomical tumor location [1,13]. Shin et al. similarly reported that orbital invasion is strongly associated with advanced T-stage disease at presentation [13].

Orbit-sparing surgery was performed in 76.81% of cases, while orbital exenteration was required in 23.19%. This reflects a contemporary shift toward conservative orbital management, supported by accumulating evidence that orbital preservation can be achieved without compromising oncological principles in appropriately selected patients [5,7]. Lisan et al. reported comparable preservation rates with similar oncological outcomes in selected cases [5], while Ferrari et al. highlighted the role of multimodal strategies in improving resectability and orbit preservation [12].

Earlier practices favored routine exenteration; however, current literature suggests that orbital removal should be reserved for cases with extensive involvement of critical orbital structures, as limited orbital invasion does not always worsen prognosis [14]. This change reflects advances in imaging, endoscopic techniques, and multidisciplinary treatment planning.

Statistical analysis demonstrated a significant difference in mean age across histological subtypes (p = 0.003), with neuroectodermal tumors such as olfactory neuroblastoma occurring in younger patients and salivary gland-type and rare tumors occurring in older patients. This reflects known biological differences among sinonasal malignancies, where distinct histologies follow different oncogenic pathways [1,10].

No significant association was observed between histological subtype and gender distribution (p = 0.92), indicating that sex is not a major determinant of tumor type in sinonasal malignancies with orbital involvement. This is consistent with prior studies showing a relatively uniform gender distribution across histological categories [10].

Similarly, no significant association was found between histology and stage at presentation (p = 0.41), as the majority of patients presented with stage IV disease across all tumor types. This reinforces the well-recognized pattern of late presentation in sinonasal cancers due to delayed symptom recognition and complex sinonasal anatomy [1,13,15].

The pattern of orbital invasion was also not significantly associated with histological subtype (p = 0.95), suggesting that orbital spread is largely governed by anatomical pathways rather than tumor biology. Imaging-based studies have similarly demonstrated that lamina papyracea erosion is the most common and earliest route of orbital extension regardless of histology [6,12].

Treatment modality also showed no significant association with histological subtype (p = 0.34), indicating that management decisions are primarily influenced by anatomical extent and resectability rather than tumor histology alone. Contemporary series similarly support standardized multimodal treatment approaches across histologies based on disease stage and local invasion [5,7,14].

However, a significant association was observed between histological subtype and surgical approach, particularly regarding orbital exenteration (p < 0.001). More aggressive histologies showed relatively higher rates of orbital sacrifice, reflecting increased local invasiveness and deeper orbital compartment involvement. Prior studies have similarly emphasized that the need for exenteration is more closely related to the extent of orbital involvement than histological subtype alone [5,14].

Clinically, ocular symptoms were common, with ocular pain, proptosis, and epiphora being the most frequent findings. These manifestations are consistent with published literature showing that orbital invasion often presents with multiple concurrent ocular symptoms, reflecting advanced local disease at diagnosis [4,13].

Overall, this study highlights that sinonasal malignancies with orbital involvement are typically diagnosed at an advanced stage, with squamous cell carcinoma being the most common histology. Orbital invasion most frequently occurs through the lamina papyracea and is associated with significant ocular symptom burden. Management is increasingly focused on orbit preservation where feasible, supported by advances in imaging, surgical techniques, and multidisciplinary care [5,7,12]. Early detection and coordinated treatment planning remain essential for optimal outcomes in this aggressive disease spectrum. This study provides a detailed analysis of 78 cases of sinonasal malignancies with orbital involvement, with standardized clinicopathological evaluation across histological subtypes. The retrospective design and single-center setting may limit external generalizability. In addition, absence of long-term follow-up precluded assessment of outcomes such as survival and recurrence.

## Conclusions

Sinonasal malignancies with orbital involvement are most commonly diagnosed at an advanced stage, with the majority of patients presenting with stage IV disease. Squamous cell carcinoma was the predominant histological subtype, and the lamina papyracea was the most frequent pathway of orbital extension. A significant difference in mean age was observed across histological subtypes. In contrast, no significant association was found between histological subtype and gender distribution, stage at presentation, pattern of orbital invasion, or overall treatment modality. However, histological subtype showed a significant relationship with the type of surgical management, particularly the need for orbital exenteration. Clinically, symptoms such as ocular pain, proptosis, and epiphora were commonly encountered, reflecting advanced local disease at the time of presentation.

## References

[1] Dulguerov P, Jacobsen MS, Allal AS, Lehmann W, Calcaterra T (2001). Nasal and paranasal sinus carcinoma: are we making progress? A series of 220 patients and a systematic review. Cancer.

[2] Bhattacharyya N (2003). Factors affecting survival in sinonasal malignancy. Arch Otolaryngol Head Neck Surg.

[3] Lund VJ, Wei WI (2015). Endoscopic surgery for malignant sinonasal tumours: an eighteen year experience. Rhinology.

[4] Turri-Zanoni M, Lambertoni A, Margherini S (2019). Multidisciplinary treatment algorithm for the management of sinonasal cancers with orbital invasion: A retrospective study. Head Neck.

[5] Lisan Q, Kolb F, Temam S, Tao Y, Janot F, Moya-Plana A (2016). Management of orbital invasion in sinonasal malignancies. Head Neck.

[6] Salfrant M, Garcia GC, Guichard JP (2021). Imaging of skull base and orbital invasion in sinonasal cancer: correlation with histopathology. Cancers (Basel).

[7] Ganly I, Patel SG, Singh B (2005). Craniofacial resection for malignant paranasal sinus tumors: report of an International Collaborative Study. Head Neck.

[8] Castelnuovo P, Lepera D, Turri-Zanoni M (2013). Quality of life following endoscopic endonasal resection of anterior skull base cancers. J Neurosurg.

[9] Nicolai P, Battaglia P, Bignami M (2008). Endoscopic surgery for malignant tumors of the sinonasal tract and adjacent skull base: a 10-year experience. Am J Rhinol.

[10] Mahalingappa YB, Khalil HS (2014). Sinonasal malignancy: presentation and outcomes. J Laryngol Otol.

[11] Amin MB, Edge SB, Greene FL (2017). AJCC Cancer Staging Manual. 8th ed. CA Cancer J Clin.

[12] Ferrari M, Migliorati S, Tomasoni M (2021). Sinonasal cancer encroaching the orbit: ablation or preservation?. Oral Oncol.

[13] Shin CH, Lee HJ, Chung YS, Kim JH (2022). Treatment outcomes of sinonasal malignancies involving the orbit. J Neurol Surg B Skull Base.

[14] Safi AF, Behn L, Rothamel D (2017). Therapy of sinonasal malignancies invading the orbit-orbital exenteration versus preservation of the orbit plus radiotherapy. J Craniomaxillofac Surg.

[15] Anschuetz L, Hohenberger R, Kaecker C, Elicin O, Giger R, Caversaccio M (2023). Sinonasal malignancies: histopathological entities, regional involvement and long-term outcome. J Otolaryngol Head Neck Surg.

